# Social capital and community resilience to socio-natural hazards in informal settlements of Chile’s extractive north: city of Copiapó, Atacama region, Chile

**DOI:** 10.3389/fpubh.2026.1914169

**Published:** 2026-09-08

**Authors:** Yasna Contreras, Mariana Pérez

**Affiliations:** 1 Department Of Geography, University of Chile (FAU), Santiago, Chile; 2 El Colegio de Mexico AC, Santa Teresa, Mexico

**Keywords:** Chile, community resilience, disaster risk reduction, extractive cities, informal settlements, mass movement, social capital

## Abstract

Informal settlements in Chile’s extractive cities concentrate structural vulnerability and exposure to multiple socio-natural hazards, including mass movement processes, flooding, and fires, among others. The article asks: how do different forms of social capital translate into differentiated capacities for resilience (coping, adaptive, transformative) in the face of the hazards confronting informal settlements in the mining city of Copiapó, Atacama Region? The discussion combines research methods applying semi-structured interviews with community leaders and institutional actors, and participatory mapping workshops aimed at identifying meeting points in the event of mass movement risk. Three findings emerge. First, bonding capital mobilizes labor, information, and resources—housing committees, early-warning networks, and communal soup kitchens—that sustain the response to hazards, but do not compensate for structural deficits in infrastructure and services. Second, linking capital distributes institutional access asymmetrically: settlements that are formally registered and hold active concessions specifically gain access to mitigation and regularization works, while unregistered settlements remain exposed, which explains why communities with similar levels of internal cohesion achieve markedly different adaptive capacities. Third, intersectoral coordination shows transformative potential with respect to mass movement risk, but remains underdeveloped for other hazards given bureaucratic fragmentation and the mismatch between institutional timelines and urgent needs. Community resilience thus emerges as a negotiated, culturally situated process, in which internal solidarity, gendered networks, and territorial belonging interact with—and are obstructed by—the institutional frameworks that govern access to disaster risk reduction resources. The policy implications point toward equity-sensitive approaches to linking capital that reduce asymmetries in institutional access without instrumentalizing community self-management.

## 1 Introduction

More than one billion people currently live in informal settlements worldwide, occupying territories that expose them to multiple socio-natural hazards. Far from constituting exceptional or transitory spaces, informal settlements represent a structural expression of contemporary urban inequalities and of the ways in which urban growth systematically displaces low-income groups toward environmentally vulnerable territories (1). Disaster risk cannot be understood solely as a product of exposure to socio-natural hazards; it is a socially produced condition that challenges the adaptive capacities, resilience, and governance frameworks of both communities and institutions. This, in turn, raises the question of how awareness can be built among communities that everyday risk mitigation and collective inter-institutional coordination themselves constitute strategies that produce communities better prepared to face any type of hazard.

The specific mechanisms through which preventive, participatory, and community-based approaches can be incorporated into risk governance in informal settlements remain insufficiently specified, and existing studies rarely delimit whether their principal analytical focus is social capital, community resilience, multi-hazard exposure, urban informality, or institutional risk governance as such. This article addresses this gap by centering its analysis on social capital and community resilience, and does so, unlike previous studies of Chilean cities, by specifying the concrete causal mechanisms—rather than merely the general association—through which each form of social capital translates into differentiated capacities for coping, adaptation, and transformation. The analysis is examined empirically through hazards linked to mass movement processes in the Andacollo sector of the city of Copiapó, a peripheral territory with a high concentration of informal settlements. In this space we identified mass movement, structural fire—given the materiality of housing—health risk associated with the absence of sanitation, and soil contamination due to the proximity of mine tailings as the principal hazards. However, we focus the discussion exclusively on mass movement processes, as these are the hazards of greatest impact and recurrence in the city of Copiapó.

The existing literature on Copiapó following the 2015 and 2017 flash floods has developed mainly along three strands, without an integrated treatment of social capital and community resilience in informal settlements. A first line of research analyzes how hydrometeorological and geomorphological conditions were determinant in the disaster associated with the 25 M flash flood of 2015, and how the historical recurrence of events in the region’s ravines explains the flooded area within the city, the deaths, and the social and economic losses (2, 3). Castro et al. (4) examine specifically the hydromorphological socio-natural hazards affecting the city of Copiapó, offering a territorial analysis that precedes and complements the approach of this article on mass movement.

A second, psychosocial and educational line of research examines how people cope with flash floods, and what the effects of post-traumatic stress and community post-traumatic growth were among the population affected by the 2015 and 2017 events. Some of this research employs mediational models centered on the subjective experience of risk (5, 6). Pérez et al. (7) document how certain educational institutions in the region developed specific disaster resilience capacities following the 2015 flash flood, underscoring the role of school communities as spaces for rebuilding the social fabric.

A third strand has analyzed the sociopolitical dynamics of the post-disaster recovery process, emphasizing the dispute over urban space rather than community mechanisms of resilience (8). None of these lines examines informal settlements in Copiapó specifically, nor specifies the mechanisms through which bonding, bridging, and linking social capital translate into community and transformative resilience in these territories. Recent research on informal settlements in other Chilean cities has begun to propose that resilience should be read through the notion of dignity and from the subaltern voices of communities, rather than from ideas of stability or a return to a prior equilibrium (9), but without equivalent studies in the extractive and flood-prone context of Copiapó.

The three lines through which Copiapó has been studied make clear that a disaster such as the 2015 flash flood in Copiapó demands the articulation of scientific-technical knowledge with social, community, and ancestral knowledge. Several researchers from the Universidad de Chile conclude that resilience in the Atacama region and in Copiapó does not constitute an isolated reaction to the 2015 event; rather, it is constituted as a cultural and historical disposition of desert communities to repeatedly overcome adverse conditions (recurrent flash floods since the nineteenth century, geographic isolation, institutional abandonment) (3). This suggests thinking of resilience as a capacity sedimented in memory and in the social organization of the territory over time.

This capacity sedimented in memory and in the territory’s social organization today demands an analysis of how informal settlements in Chile require rethinking public policy, both in housing and in risk regulation and zoning. The complexity stems from the diversity of typologies of informal settlements produced in urban territories, in urban-forest and urban-agricultural interfaces, and in rural areas. All of these have emerged forcefully in the wake of the COVID-19 pandemic as a response to processes of housing commodification, whether through rental or purchase markets (10).

In Chile, as of 1996 there were almost 972 informal settlements inhabited by 115,000 families (11). A large share of these settlements were located in urban areas of intermediate cities and the capital; however, all were located in areas exposed to multiple risks. By 2024–2025, this figure had risen to 1,428 settlements and more than 120,000 families (12). Specifically in the Atacama Region, where the principal city of Copiapó is located, almost 150 settlements are recorded, home to more than 10,000 families, both Latin American migrants and Chileans. Copiapó is the third Chilean commune with the largest number of settlements (6.7% of the national total). Against this backdrop, the article analyzes the forms of social capital and community resilience developed in informal settlements exposed to mass movement risk in the city of Copiapó, Atacama Region. Drawing on a qualitative and territorial approach, the study examines how communities build adaptive capacities in the face of risk, and how these are articulated with—or come into tension with—institutional responses to disaster risk reduction. Rather than assuming this relationship, the study proposes it as an analytical hypothesis: that community resilience in informal settlements is a negotiated, relational, and politically contested process, conditioned by the asymmetric distribution of linking social capital and by the structural limits of inter-institutional coordination in contexts of urban informality.

## 2 Theoretical discussion: the social production of risk in informal settlements

Analyzing informal settlements as spaces of production and accumulation of disaster risk requires a conceptual architecture that articulates at least three theoretical dimensions: the social construction of risk together with the dynamics of urban informality; social capital as a resource for collective management and a strategy that fosters risk awareness; and community resilience as the capacity for transformation once communities become aware of the risks they face and develop collective means to address them. These concepts are deeply intertwined: disaster risk is produced and unequally distributed across informal territories; social capital determines communities’ capacity to mobilize resources in times of crisis; and resilience emerges—or is obstructed—depending on how social networks articulate with the institutional frameworks that govern the territory.

Understanding of disaster risk has undergone a profound conceptual transformation since the second half of the twentieth century. The dominant view—which attributed disasters to the exceptionality of natural phenomena—was systematically challenged by a perspective that reframes them as products of historically constructed social, political, and economic conditions. Since Hewitt (13), this paradigm has shifted the analytical focus from the external physical agent toward the structures of vulnerability that amplify its effects, a position later systematized by Blaikie et al. (1) through the pressure-and-release model, which links root causes—exclusionary economic systems, unequal access to power—with dynamic pressures and unsafe conditions to explain the differential production of risk in a territory.

The production of knowledge on disaster risk in informal settlements has traditionally been dominated by technocratic approaches that privilege quantitative modeling and the physical analysis of hazards, marginalizing the territorial knowledge of exposed communities (14). This epistemic hegemony reproduces asymmetries of power in defining what constitutes valid knowledge about risk and who is authorized to produce it, rendering invisible the experiences, practices, and knowledge that inhabitants of vulnerable territories have developed through their everyday dwelling (15). In informal settlements of the Global South, these epistemic inequalities translate into risk management systems that reproduce structural exclusions. Urban informality thus constitutes not only a legally irregular condition but also a differentiated form of territorial exposure to risk, where the absence or fragmentation of institutions conditions the possibilities for prevention, mitigation, and community preparedness (16).

Lavell (17) and García (18) deepened this perspective by incorporating the historical and political dimensions of the construction of risk: disasters are not discrete events but the cumulative expression of decisions about land use, the distribution of public investment, and development models that exclude broad sectors of the population. The Sendai Framework for Disaster Risk Reduction 2015–2030 (19) has institutionalized globally the notion of risk as a social construction, promoting preventive, participatory, and multisectoral approaches that go beyond the reactive management of emergencies. In informal settlements, risk is not an element external to the condition of informality but one of its constitutive dimensions: it is produced in the territory, accumulated in bodies, and reproduced through the very structures that generate housing exclusion (Cannon, 2008).

Informal settlements are conceptualized as spaces that emerge outside the planning system, under conditions of precariousness and lacking the elements that characterize formally constituted neighborhoods. They are associated with “irregular land tenure, self-built housing, low levels of infrastructure, and low-income residents” [(20), p. 119]. Beyond these physical descriptions, informal settlements must be understood as spaces of concentrated exposure to multiple hazards, where diverse and vulnerable populations exist on the margins of public policies and regulatory frameworks that govern risk only within formally delimited urban boundaries (16). The production of community resilience therefore requires thinking about risk beyond regulatory boundaries, incorporating territories historically excluded from preventive and mitigation policies (17), regardless of whether they are located within an urban area, at the urban–rural interface, or in agricultural and forest zones (21).

Along these lines, social capital constitutes one of the most productive and debated concepts in the contemporary social sciences. Its three foundational theorizations—Bourdieu (22), Coleman (23), and Putnam (24)—share the recognition that resources mobilizable through social networks constitute a form of capital as real as economic or cultural capital, although they diverge in their analytical emphases. The literature on social capital distinguishes at least three structurally differentiated forms, whose distinction is not merely taxonomic but responds to distinct causal mechanisms of collective resource production. Bonding capital refers to strong, dense, and homogeneous ties between socially and culturally similar actors—families, neighbors, co-ethnic networks—which Putnam (25) characterizes as the social glue capable of generating immediate reciprocity and short-term mutual support, but which, precisely because of its internal density, can lead to group closure and the exclusion of those who do not belong to the network. Bridging capital, by contrast, connects heterogeneous actors through weaker but horizontal ties (organizations or diverse sectors within the same territory), whose effectiveness rests on the strength of weak ties (26), in the circulation of novel, non-redundant information among groups that would otherwise remain disconnected. In contrast to both horizontal forms, linking capital introduces a vertical and asymmetric dimension of power: it describes the capacity of individuals or communities to access institutions, authorities, or agents with resources and formal attributions that exceed what the community network itself can mobilize. Szreter and Woolcock (27) coined this third concept precisely because they observed that the bonding-bridging pairing was insufficient to explain why communities with similar levels of internal cohesion exhibited profoundly different outcomes in their access to health services and state resources. The explanatory factor lay in their differing capacity for vertical connection with the institutional apparatus. This conceptual triad projects directly onto resilience theory through the work of Woolcock and Narayan (28), who explicitly links each form of social capital to a differentiated capacity—getting by, getting ahead, and getting out of precariousness or organizing—thereby establishing a direct theoretical antecedent for thinking about how these three forms not only coexist but produce qualitatively distinct resilience trajectories.

In contexts of urban informality, Portes (29) and Woolcock and Narayan (28) argue that social capital can operate both as a resource for resilience and as a mechanism for the reproduction of exclusions: dense networks in informal settlements provide solidarity and resources amid precariousness, but can also generate dependencies, reproduce internal hierarchies, and limit the access of the most vulnerable members to external opportunities. Aldrich (30) and Aldrich and Meyer (31) demonstrate empirically that communities with greater social capital—especially institutional linking capital—recover more quickly from disasters and with smaller losses than those that rely solely on internal resources or external state support.

Community resilience is configured as a conceptual tool for understanding how communities confront, adapt to, and transform conditions of crisis and vulnerability. From a public health perspective, Norris et al. (32) define community resilience as a process of positive adaptation that emerges from the interaction of four primary capacities: economic resources, social capital, information and communication, and community competence. In informal settlements, these capacities are deeply conditioned by historical patterns of territorial precariousness and urban exclusion, but are also limited or enabled by the ties established with institutions at the local or proximate scale. Resilience, in its most general sense, refers to the capacity of a system, community, or individual to absorb disturbances, adapt, and continue functioning (33, 34). Community resilience comprises a process linked to local agency, territorial knowledge, and supportive institutional infrastructure (35). It is also understood as a community’s capacity to mobilize resources in order to respond to change, developmental strains, and crises (36).

Transformative resilience, by contrast, describes a distinct and more demanding type of capacity: that of modifying the structural, political, and institutional conditions that produce vulnerability in the first place, rather than merely absorbing its effects or adapting to them (37, 38). Folke et al. (33) similarly distinguish between persistent, adaptive, and transformative resilience, noting that the latter implies the creation of a fundamentally new system when existing ecological, economic, or social conditions become unsustainable. Applied to informal settlements in extractive cities, this framework allows empirical distinction between community practices that reduce everyday exposure to risk without altering the structural causes of territorial precariousness—forms of coping and adaptive resilience—and those interventions, coordination efforts, or political disputes capable of modifying access to land, infrastructure, and institutional disaster risk reduction resources—properly transformative resilience [Pelling, 2003; (39)]. In sum, when read through community resilience and its link to the three types of social capital, resilience in informal settlements cannot be reduced to internal cohesion or neighborly solidarity, but must be examined as a relational process that depends on the articulation between collective capacities and the institutional frameworks that enable or constrain their deployment (40).

## 3 Methods

The study focuses on the Andacollo sector of Copiapó, capital of Chile’s Atacama Region and an emblematic extractive city whose recent urban growth has been driven predominantly by copper and gold mining. The methodological strategy combined participatory instances of collective data production with semi-structured interviews, following a sequential methodological triangulation design that allowed the territorial readings of residents, community leaders, and institutional actors to be contrasted with one another. Three workshops were held with a cumulative participation of approximately 60 residents (October 2024 and October 2025), designed under the principles of participatory social mapping and participatory action research. A first workshop addressed territorial delimitation and mapping of the internal organization of housing committees, which also made it possible to identify the leaders who sustained the process. A second workshop on collaborative recognition of socio-natural hazards was held together with the Fire Department and Police, focused on mass movement given the impossibility of addressing multiple hazards within a limited number of sessions. A third workshop aimed to validate, together with the community and institutions (MINVU Atacama, the Municipality of Copiapó, and SERVIU’s Precarious Settlements Department), meeting points in the event of mass movement.

For the individual interviews, purposive sampling oriented toward maximum variation—rather than statistical representativeness—was applied. The Andacollo sector has approximately 2,000 residents distributed across 17 housing committees, of which 6 concentrate most of the organizational activity. All leaderships, both validated and non-validated, were invited to participate, resulting in interviews with 10 leaders from four committees in total. This recruitment strategy responded to a deliberate methodological decision: the coexistence of validated and non-validated leaderships—and the dispute over legitimacy between them—constitutes in itself a relevant dimension of the phenomenon under study, rather than noise to be filtered out of the sample (41). Of the 10 leaders interviewed, 6 were Bolivian men over 50 years of age and 4 were Chilean women between 35 and 55 years of age. Most leaders have lived in the Andacollo sector for more than 3 years and, on average, for more than 10 years in the city of Copiapó overall. During the go-along walks, women leaders reported recurrent conflicts with other leaders, specifically Bolivian migrants linked to informal urbanization practices and the arbitrary allocation of building plots. This tension was documented as a finding rather than resolved through the sampling procedure.

Institutional actors were recruited according to their availability and genuine interest in participating, including professionals from the Regional National Disaster Prevention and Response Service (SENAPRED, 2 people); the Municipality of Copiapó (2 risk officers); the Ministry of Housing and Urban Planning (MINVU), specifically its Atacama Region Housing and Urban Planning Service units (SERVIU, Precarious Settlements, 2 people); 2 professionals in charge of the Risk Unit of SEREMI-MINVU Atacama; 1 regional representative of the National Geology and Mining Service; and 1 representative of the Directorate of Hydraulic Works (DOH) of the Ministry of Public Works (MOP).

Fieldwork was conducted between 2024 and 2025. Workshops were held in person in Copiapó, while a subset of institutional interviews (Municipality, SENAPRED, SEREMI MINVU, and SERVIU) was conducted via video call for reasons of availability. Data collection among institutional actors reached a satisfactory level of thematic saturation regarding the axes of inter-institutional coordination and risk management. In the case of community leaderships, by contrast, coverage was constrained both by the fieldwork schedule and by the actual availability and willingness to participate of the 17 existing leaderships; this limitation is explicitly declared, in line with the criteria of transparency and methodological reflexivity recommended for qualitative research in contexts of high social vulnerability and contested community representation.

The participatory mapping methodology involved residents in the collective production of their own maps, identifying everyday circulation routes, significant places, sectors perceived as dangerous or safe, neighborhood landmarks, and possible evacuation zones. This process produced four collective maps that revealed both convergences and divergences in territorial perceptions and community risk-response strategies. Go-along walks were also conducted with community leaders and residents of the prioritized settlements, who guided routes through their everyday trajectories, identified territorial obstacles, recounted experiences related to previous hydrometeorological events, and shared situated knowledge about territorial dynamics. This method enabled an embodied and territorial understanding—in the sense of knowledge produced by the body moving through space—of elements that rarely emerge through conventional cartographic analysis alone.

The information generated participatorily was systematized using Geographic Information Systems (GIS), overlaying community maps with institutional proposals and previously modeled hazard zones. This process of cartographic translation required particular attention to avoid reducing or distorting the territorial knowledge expressed by participants when adapting it to standardized technical languages (15), a tension inherent to any exercise in counter-mapping that engages with state planning instruments.

The names of all participants were protected through alphanumeric codes, in accordance with the commitments established in the informed consent documents. Data were analyzed using NVivo 15 through a mixed thematic coding process. The code tree was built through triangulation among the research team members, following consultation with the institutions. Five dimensions linking social capital and resilience were coded: (i) motives for building and self-managing housing in the informal settlement; (ii) memory of disaster risk in interviewees’ life biographies; (iii) everyday actions to address mass movement risk within and outside the home; (iv) internal and inter-institutional coordination mechanisms for addressing mass movement risk; and (v) reasons for taking on community leadership roles. Thematic saturation was assessed continuously throughout the coding process, particularly during the interview phase with community leaders and institutional actors, although the criterion was met at different points for each group, depending on the homogeneity or heterogeneity of their experiences and positions regarding risk.

In the case of community leaderships, saturation was reached at interview No. 6, which is explained by the annual recurrence of mass movement events in Copiapó, systematically triggered during the rainy season—between April and August—generating a shared and consistent experience among the leaders interviewed. Among institutional actors, by contrast, the saturation criterion was met later, at interview No. 9, a process that proved more complex to establish given the heterogeneity of positions identified across the different institutions involved. On one hand, a relatively consistent level of coordination was observed among the different MINVU departments (SEREMI, SERVIU, and Precarious Settlements), the municipality, and SENAPRED regarding ways of addressing risk. On the other hand, actors such as the MOP and SERNAGEOMIN showed divergent and at times conflicting views on how to address informal settlements in relation to risk, given that these settlements are located outside current urban regulations or regulated territorial boundaries.

## 4 Results

Informal settlements in the Andacollo sector of the city of Copiapó face two latent socio-natural hazards: mass movement and structural fire. However, this article analyzes only the former, given that the two flash-flood events (2015 and 2017) drove an unprecedented inter-institutional relationship within Chile. The city of Copiapó demands transversal analysis given its condition as an extractive mining territory, surrounded by numerous mine tailings deposits within and near the city. In the ravines of the southern part of the city, mine tailings can be observed concentrated on the upper slopes, producing a complex tension in a city encircled by the Copiapó River.

### 4.1 The City of Copiapó: processes of urban expansion and their relationship to socio-natural hazards

**Figure 1:**
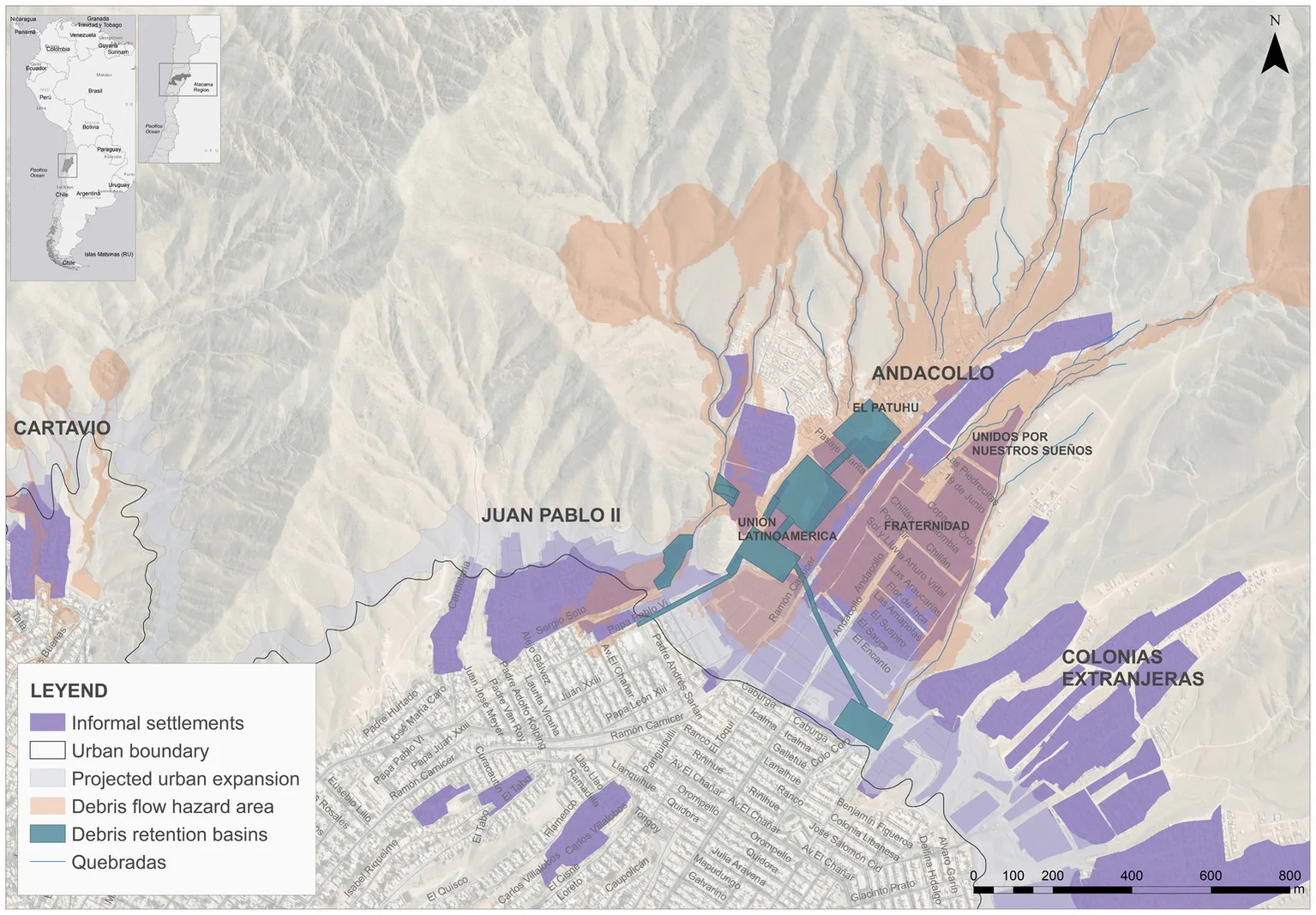
City of Copiapó, Andacollo Sector, Atacama Region source: Authors, ONG-Techo (12), Google Earth georeferencing, March 2026.

Copiapó, capital of the Atacama Region, has undergone accelerated urban growth over the past decade, marked by the expansion of informal settlements on the city’s high, western peripheries. By 2024, the municipality recorded approximately 169,000 inhabitants, concentrating around 56% of the regional population. This growth has been accompanied by a substantial increase in the foreign migrant population, which rose from 6,032 people in 2017 to more than 19,000 in 2024 (42), as well as by a sustained increase in the housing deficit and pressure on the urban land market. Informal settlements have multiplied rapidly: while the city recorded 27 settlements with 1,118 households in 2012, by 2024 there were 147 settlements housing approximately 11,000 families, many located in areas exposed to mass movement, shown in orange in Figure 1. This peripheral growth is concentrated in the Andacollo sector, which currently has more than 17 housing committees and approximately 2,000 families distributed across informal occupation polygons, shown in lilac in Figure 1.

The causes of settlement expansion cannot be understood solely through the dynamics of housing demand, but rather as an expression of structural processes linked to unequal urbanization and to the extractive model predominant in Atacama. Mining accounts for approximately 44% of regional GDP and has helped consolidate an image of economic prosperity that attracts migrant population and low-income workers. However, this same extractive economy has significantly increased the cost of living and the value of urban land, shaping a housing market oriented mainly toward mining workers and middle- and upper-income sectors. In Copiapó, apartments averaging 51–60 m^2^ are priced between approximately USD 87,000 and USD 217,000—levels structurally inaccessible to residents of informal settlements. As a result, broad sectors of the population are excluded from access to formal housing and are displaced toward precarious forms of territorial occupation. The root causes of vulnerability include a regional housing deficit of approximately 16,000 units in Atacama—with Copiapó concentrating 40% of the deficit and 52% of its housing classified as irrecoverable—combined with land speculation and subsidy levels insufficient to cover regional housing costs.

The growth of informal settlements accelerated after the 2015 and 2017 flash floods, when families displaced from flood-affected areas sought housing solutions in the city’s higher peripheries because of the severe insalubrity, water contamination, and dampness inside damaged homes. Paradoxically, these higher peripheries correspond to alluvial-fan ejection cones and drainage basins that feed rainwater retention ponds (Figure 1), making them zones of high exposure to mass movement, debris flows, and flooding. The city has a total surface area of 17,000 km^2^, with urban land limited to 1,511 hectares, severely restricting the availability of well-located sites free of exposure to flooding, mass movement, or seismic risk. Among the dynamic pressures are the 2015 flash flood as a structurally destabilizing event, the COVID-19 pandemic, which accelerated informal occupations, and sustained southward migration.

During fieldwork, permanent flows of greywater from washing and domestic activities were observed on the main circulation streets and in areas adjacent to the ravines. In numerous cases, wastewater pipes discharge directly into these drainage channels, which also function as flash-flood evacuation routes. This situation creates a scenario of double vulnerability: it increases health risks through contamination and the proliferation of vectors, while simultaneously altering geomorphological conditions by weakening slopes composed of low-compaction sedimentary material and increasing the accumulation of solid waste in potentially active channels. Read through the framework of the social determinants of health, this double vulnerability constitutes not only an environmental or infrastructural problem but a territorial expression of inequality in access to sanitation, which directly conditions the burden of transmissible and vector-borne disease faced by families in Andacollo, and which is distributed just as asymmetrically between registered and unregistered settlements as mass movement risk itself. Risk, therefore, cannot be understood solely from the location of settlements in exposed zones, but also from the everyday transformations that patterns of habitability produce upon the territorial system: the infilling of ravines, the diversion of channels, and the construction of housing over runoff zones alter the circulation of flows and complicate institutional mitigation capacities.

The legal irregularity of the settlements generates a chain of self-reinforcing exclusions: without a title of ownership, the sanitary utility cannot connect potable water; without potable water there is no sanitary feasibility; without sanitary feasibility, SERVIU cannot develop urbanization projects; and without urbanization there is no title granted. This legal trap has direct social dimensions: the absence of piped potable water means buying it from private water tanker trucks at prices ranging from USD 9 to USD 15 per 1,000 liters—five times the regulated tariff—consuming a disproportionate share of family income. A municipal official summarized one of the less visible consequences:

“The silent risk of groundwater contamination from cesspits in areas without sewerage is a phenomenon that has already caused deaths in Copiapó in the late twentieth century.” (Municipal official, Copiapó, 2024)

This testimony underscores that any intervention in Copiapó’s informal settlements requires multi-hazard policies and plans capable of simultaneously addressing visible and invisible risks.

### 4.2 Bonding social capital and situated resilience: community organization and everyday risk management

“In April we always organize through WhatsApp because we flooded three years ago. But the neighbors up above and those living near the retention pond leave everything full of trash. We know the flash flood could come, but where would we go? Here there’s no money to pay for anything.” (Male resident, Andacollo, Copiapó)

This testimony illustrates how residents perceive their exposure to hazards and risks, while at the same time recognizing the need for community organization as a form of bonding social capital capable of partially reducing the impacts of fires or mass movement processes. The possible flash flood is not the only concern; the invisible effects of reliance on septic pits without regular emptying, water supply through privately operated tanker trucks at disproportionate cost, and overcrowding in precarious and highly flammable housing constitute place-based conditions (43) that amplify exposure and limit response capacity ahead of any critical event.

Within the settlements, internal networks are organized particularly around two socio-natural hazards: flash floods, which can occur between April and July, and structural fires, given the highly combustible nature of wood-frame housing. The diverse ethnic composition of the settlements—home to Chilean, Bolivian, Peruvian, and Venezuelan families—reflects processes of intercultural integration that partially mitigate the potentially exclusionary character of inward-oriented bonding capital (27). Several Bolivian interviewees reported having previously experienced repeated displacement due to flooding and mass movement in different rural territories of Bolivia, a biographical history that simultaneously activates risk preparedness and, paradoxically, can dull risk perception in the new context by normalizing exposure.

Gender dynamics constitute a cross-cutting finding across all the settlements: women play a disproportionate and structurally unrecognized role in building and sustaining community social cohesion. Women chair housing committees and neighborhood councils, manage projects, and lead negotiations with the municipality and ministerial bodies. This deployment of social capital rests on an overload of unremunerated reproductive labor—a structural condition of the extractive economic system—insofar as it is women who sustain everyday life under conditions of precariousness. Recognizing this dimension is essential to ensuring that community resilience policies do not reproduce an instrumentalization of women’s labor without addressing the structural causes of vulnerability. This pattern is consistent with the broader critical literature warning that disaster risk reduction frameworks have historically treated gender as merely a demographic variable rather than as a structuring axis of vulnerability and capacity, obscuring how masculinized institutional spaces and feminized community care spaces are co-produced within the same risk-governance system (44). In Andacollo, women’s leadership of housing committees functioned simultaneously as a resilience asset and as an additional unremunerated burden layered onto already existing domestic and care responsibilities, underscoring that gender-responsive resilience policy must distinguish between recognizing women’s organizational leadership and relying on it as a substitute for public investment.

The COVID-19 pandemic acted as an amplifier of pre-existing vulnerabilities in these territories, simultaneously accelerating the formation of new informal settlements. The collapse of the health system in Copiapó when faced with an unregistered population.

“We had a health crisis, because the resources reaching health services are tied to people who are registered.” (DIDECO municipal official, 2024)

The account above illustrates how public health crises interact synergistically with territorial vulnerability in informal settlements, widening gaps in access to essential services for the most exposed populations (32). Resilience in the context of a health crisis therefore cannot be reduced to individual indicators of recovery capacity, but must be assessed in its collective and structural dimension: who has access to which networks, under what cultural and territorial conditions, and in what relationship to the State.

The health vulnerability of the Andacollo settlements is not limited to exposure to mass movement and to contamination associated with inadequate sanitation, but is also expressed in the climatic and material conditions of housing. Because these settlements are located in the heart of the Atacama Desert, several interviewees described facing intense cold at night and in the early morning—given the desert climate’s characteristic thermal amplitude—as well as dampness inside homes built with precarious materials that provide inadequate insulation from either the cold or the scarce but intense winter rainfall. These conditions were described by residents as an everyday source of respiratory illness, particularly among children and older adults.

This climatic vulnerability is deepened by lack of access to health services. Several women interviewed reported living with older adults with reduced mobility who cannot travel to the nearest health centers, given that the settlements are located in high, hard-to-access sectors, without public transport or paved routes connecting to Copiapó’s public health network. This burden falls once again on women, who take on the daily care of family members with reduced mobility as an unremunerated extension of the community role already documented above.

### 4.3 Participatory mapping and planning of assembly points for mass movement risk

**Figure 2:**
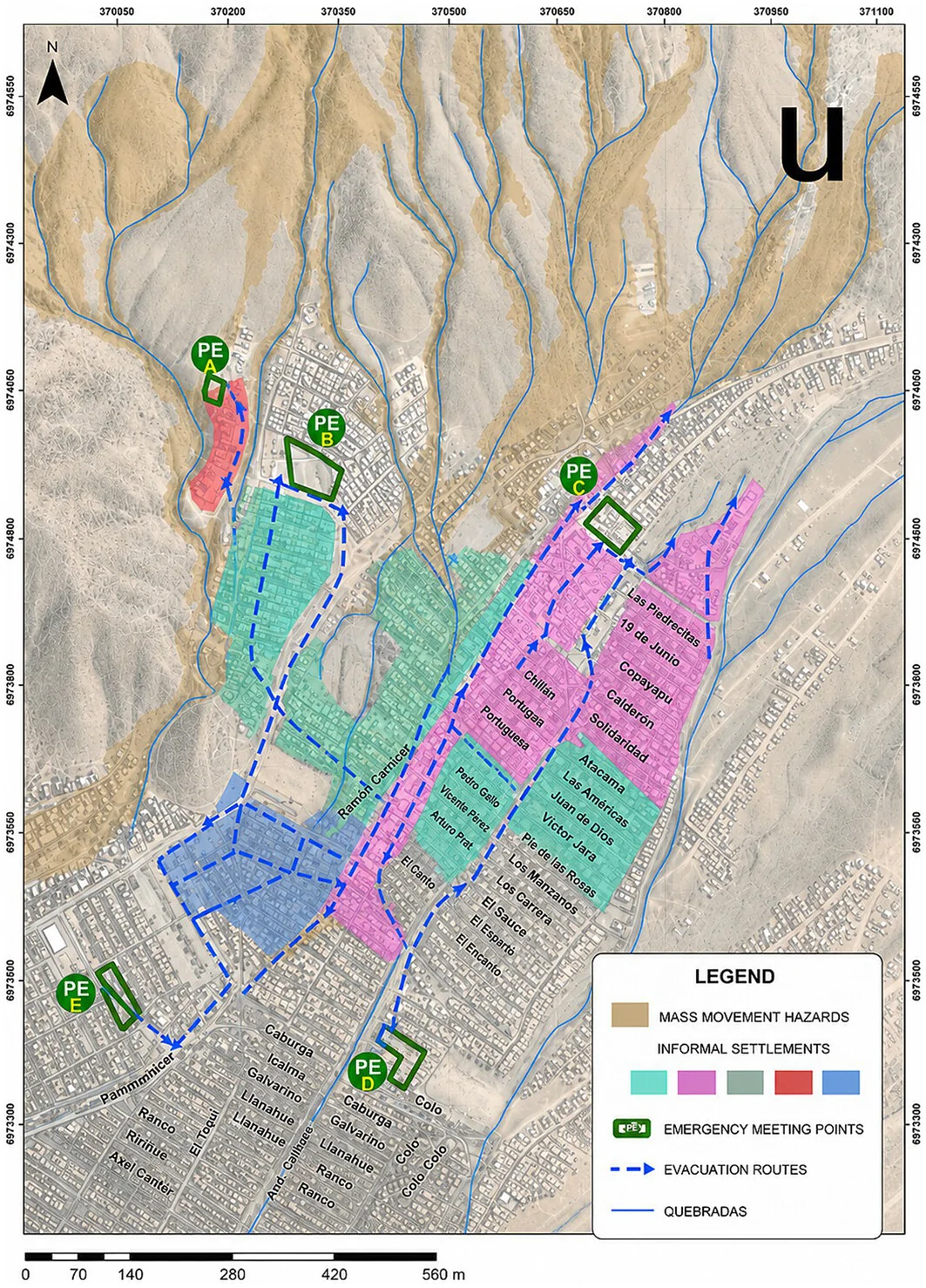
Andacollo Sector|City of Copiapó|Assembly points for mass movement risk. Source: The authors together with MINVU, SERVIU, SENAPRED, the Municipality of Copiapó, and the community, 2024–2025.

In order to address the inter-institutional and community gaps represented in Figure 2, since 2025 and as a consequence of the 2015 and 2017 flash floods, the Ministry of Housing and Urban Planning has built an alliance with SENAPRED, the Municipality of Copiapó, and academia, in order to advance toward transformative resilience with respect to mass movement risk. This alliance does not sidestep other hazards present in the area; however, advancing toward the definition of assembly points for mass movement events could constitute a first effort to bring institutions together, identify challenges, and integrate communities living in informal settlements that fall outside regulatory and legal frameworks.

Together with the community and institutions of the Atacama Region (MINVU, the municipality, and SENAPRED), areas exposed to flash floods, sediment transport, flooding, and rockfall were identified. Based on this characterization, direct and indirect risks were estimated, considering possible human and material losses. The modeling took as its reference the 100-year return period scenario (RP100), which represents the event of greatest potential impact in terms of area affected and number of exposed households. Under a precautionary logic, this scenario guided the identification of relatively safe zones and possible assembly points in the event of extreme events. The Andacollo sector benefits from structural mitigation works developed by the DOH (settling ponds), designed to contain and channel potential flash-flood flows.

Figure 2 shows the five assembly points identified within the Andacollo sector (PEA, PEB, PEC, PED, and PEE). In blue, yellow, greenish, and purple, the housing committees for which assembly points were defined in the event of mass movement are zoned. The dashed blue line identifies the best evacuation routes, although work with older adults and people with disabilities requires a different kind of approach that this study could not undertake. Fieldwork revealed multiple obstacles along the routes to the assembly points: ravines that must be crossed during hydrometeorological events, steep slopes that hinder the mobility of older adults, narrow stretches alongside precipices, unlit sectors perceived as unsafe by women, and trash accumulation that blocks circulation. Significant tensions emerged between institutional timelines and community dynamics: while institutions sought to define assembly points quickly in line with bureaucratic deadlines, communities required longer periods of deliberation, appropriation, and trust-building. During the workshops, several participants expressed distrust of institutional proposals, associated with prior experiences of broken promises, evictions, or interventions disconnected from the territory’s real needs. The construction of the assembly points was not oriented toward finding ideal evacuation solutions—practically nonexistent in a territory crossed by multiple hazards—but toward collectively identifying spaces that would reduce risk and save lives in mass movement scenarios.

**Figure 3:**
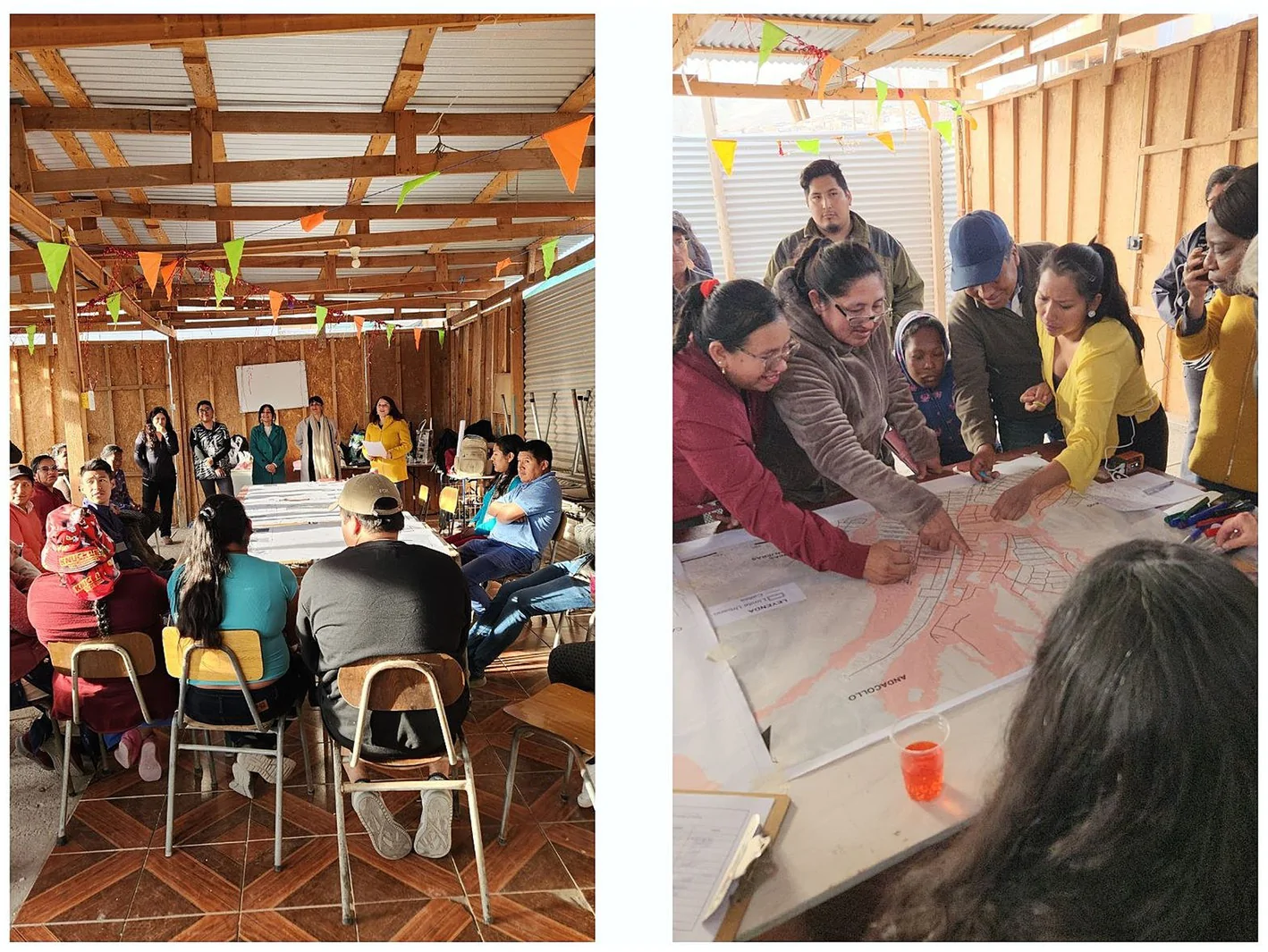
**(a, b)** Participatory mapping with Copiapó residents to identify assembly points. Source: Authors, October 2025.

The definition of the assembly points (Figure 2) was developed through a participatory process involving communities, researchers, and institutions, considering three main criteria. First, the potentially exposed population was estimated using MINVU’s Precarious Settlements cadastral data updated to 2024. Second, the minimum area required for each assembly point was calculated following the MINVU standard of approximately 3 m^2^ per person, to guarantee minimum conditions of temporary shelter. Third, travel times were assessed, with particular attention to older adults and people with reduced mobility (Figure 3a). The identified points were discussed and collectively adjusted through participatory workshops with residents of the Andacollo sector (Figure 3b). These workshops resolved cartographic questions, identified territorial obstacles, and strengthened community organizational mechanisms that were already operating informally through WhatsApp groups in response to flash-flood and fire hazards.

The interviews reveal that defining hazards does not guarantee safety in the face of multiple vulnerabilities. First, the evidence shows that the very expansion of informality can erode community disaster risk reduction resources that have already been built. One community leader stated that the assembly point assigned to her committee posed an emerging risk given the installation of new homes and families that were not part of the original grouping: “We had a [safe zone] and they covered it with a house, and there are no more empty spaces left, we have no safe spaces.” The leader described how neighboring committees that arrived later filled the ravines that previously drained rainwater with earth and rubble, degrading the drainage capacity of the entire sector and not only their own. This finding introduces a tension that the literature on bonding social capital tends to underestimate: the solidarity and organization of one committee does not guarantee safety against the occupation decisions of neighboring committees, such that one sector’s adaptive resilience can be actively eroded by another’s everyday coping resilience, within the same territory and under the same regime of informality.

Second, the same community leader explicitly distinguished between types of hazards when describing neighborly solidarity: while fires elicit an immediate, generalized collective response, security threats—shootings and gunfire—do not activate the same mutual aid, only WhatsApp alert communication and contact with police. This suggests that bonding social capital does not operate uniformly across every hazard, but that its activation depends on the specific type of risk, a specificity that studies of social capital and disasters rarely differentiate (31).

### 4.4 Linking social capital: institutional asymmetries

The construction of community resilience in Copiapó’s informal settlements depends critically on the quality and density of linking social capital established with state institutions and regulatory bodies. This type of capital—defined by vertical relationships between actors with differing institutional power—is decisive in enabling structurally vulnerable communities to access resources that cannot be mobilized internally. In the studied context, the Directorate of Hydraulic Works (DOH), the Superintendency of Sanitary Services (SISS), MINVU/SERVIU, and the Municipality of Copiapó make up the institutional map that determines which communities gain access to mitigation works, potable water, regularized electricity supply, and sanitation, urbanization, and relocation plans. Without this linking capital, as illustrated by the contrast between settlements with active municipal concessions and those left in administrative limbo, communities remain exposed to an accumulation of risks that no level of bonding capital can offset.

In Copiapó, the greatest concentration of settlements is found in the Andacollo sector (Figure 1), half of which will be incorporated into urban regulation through the extension of the urban boundary. To date, only settlements registered before 2019 (almost 60%) will benefit from housing subsidies, leaving the rest in a state of institutional uncertainty. Relocation toward the southern part of the city is under study, although those areas are also exposed to soil contamination from mine tailings and to mass movement hazards. This illustrates the complexity of relocation in a mining region where—in a structural paradox—extractive companies capture economic rents but do not invest proportionally in works that mitigate the exposure of communities and workers to multiple disaster risks.

Intersectoral coordination reveals both the potential and the limits of linking capital for transformative resilience. The DOH, under the Ministry of Public Works, coordinates with MINVU on the relocation of families from flash-flood retention-pond zones and has built containment dikes within the Andacollo sector (Figure 1). The SISS works with the sanitary utility to extend its operational territory when SERVIU formalizes new settlements. The municipal urban advisor identifies silent risks—groundwater contamination, electrical overloads, accessibility problems—that no single institution can address alone. This type of intersectoral coordination is consistent with what Keck and Sakdapolrak (45) call adaptive capacities, that is, the capacity of social systems to reorganize resources and respond to changing conditions. This has, so far, made it possible to prevent large-scale flooding in the settlements studied. However, this coordination has important limits with respect to structural fires and the various problems of overcrowding, lack of lighting, and exposure to contaminants faced by the area.

## 5 Discussion of results

### 5.1 Asymmetries of linking capital and institutional fragmentation

**Figure 4:**
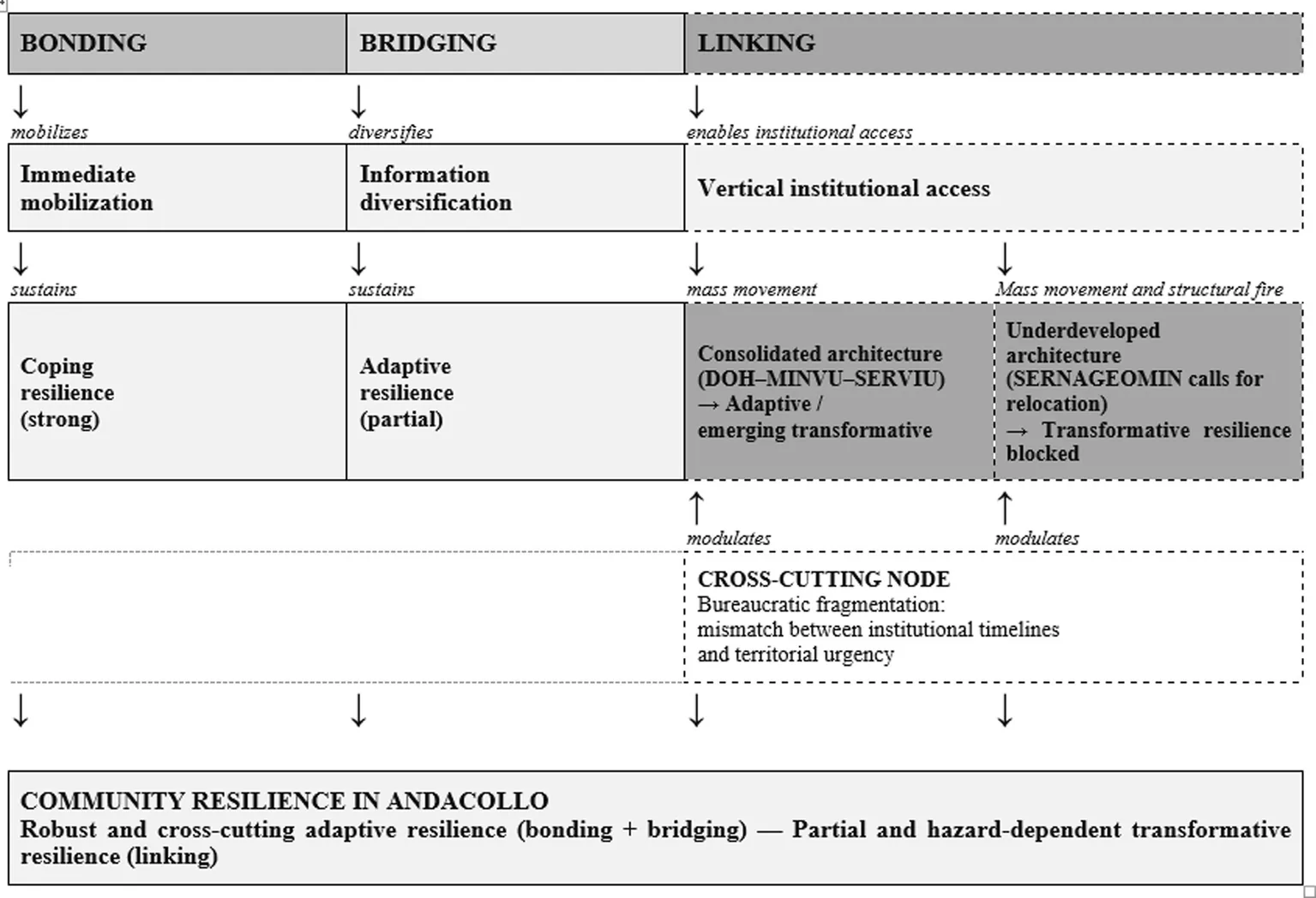
Conceptual-empirical matrix of the form of social capital and resilience in informal settlements of the city of Copiapó Source: Authors, based on analysis of interviews with leaders and institutions in the Atacama Region.

Figure 4 is configured as a conceptual-empirical matrix that relates the form of social capital identified from the interviews to the resilience capacity of the communities interviewed. The analysis of the interviews exposes the structural limits of institutional articulation in translating into effective resilience for communities. The Superintendency of Sanitary Services (SISS) lacks the legal authority to intervene outside its concession territory, leaving irregular settlements outside the potable water regulatory framework until they obtain a title of ownership. The Directorate of Hydraulic Works (DOH) must navigate environmental impact assessments, expropriation processes, and addenda to the Environmental Assessment System (SEA) that, together, delay the materialization of critical mitigation works for years. This institutional bureaucracy corresponds precisely to what Aldrich and Meyer (31) identify as one of the main barriers to building resilience in vulnerable communities: when linking social capital exists only at a declarative level, but regulatory frameworks fragment responsibilities and delay responses, communities continue to absorb the impact of risk without state resources effectively reaching their territory.

Bonding and bridging capital (Figure 4) remain invariant with respect to the type of hazard because their mechanisms—immediate mobilization and diversification of information—depend on the density of community relationships rather than on external institutional architecture (SENAPRED). Linking capital, however, does depend on institutional architecture, especially that of MINVU and SENAPRED, and it is here that the model branches: in the face of mass movement, consolidated coordination among SENAPRED, MINVU, and SERVIU brings the community closer to the transformative threshold, whereas in the face of structural fire, the absence of an equivalent coordination table keeps that same linking capital trapped below the threshold. The branch relating to mass movement processes fractures particularly around the MOP-DOH, insofar as this agency assumes, from its own role, that the construction of three settling dikes is sufficient infrastructure to cushion potential mass movement risk. Conflict emerges when communities occupy the dikes or leave trash in them. SERNAGEOMIN is an actor that does not enter into dialogue with the other institutions, insofar as it instead suggests relocating all families living in informal settlements.

The bifurcation in Figure 4 proposes that the threshold of transformative resilience is not a fixed property of the type of social capital, but a joint function of linking capital and the institutional architecture previously built for each particular hazard. Put differently, the theoretically relevant question is not only how much linking capital a community possesses, but whether that linking capital has already been institutionalized—through protocols, coordination tables, or infrastructure—for the specific risk being faced. This refines Aldrich and Meyer’s (31) argument about regulatory fragmentation as a generic barrier to resilience, specifying that such fragmentation does not operate uniformly across every hazard within the same territory, but can coexist with consolidated coordination architectures for one hazard and be absent for another, within the same community and under the same institutional regime.

The cross-cutting node of bureaucratic fragmentation, common to both branches, fulfills an additional theoretical function: it explains why not even the more favorable branch—mass movement—manages to complete the crossing into fully transformative resilience, but instead remains in a state of “emerging transformative” resilience. This is consistent with Manyena's (39) distinction between the capacity to recover and the capacity for structural change, and suggests a direct policy implication: extending to structural fire the same inter-institutional coordination architecture already available for mass movement would not replace the need to resolve cross-cutting bureaucratic fragmentation, but it would move the community closer to the threshold for both hazards simultaneously, rather than for only one. Indeed, the inter-institutional architecture relating to mass movement requires greater openness from SERNAGEOMIN in order to advance toward transformative resilience.

Communities in the informal settlements deploy substantial adaptive capacities sustained mainly by bonding social capital, expressed in the internal coordination of housing committees, the use of WhatsApp groups for alerts and communication, and the collective organization of ravine cleaning and waste management during the months of highest flash-flood risk. These practices demonstrate forms of popular risk management (Maskrey, 1989), through which communities develop technical knowledge and organizational strategies in response to everyday hazards:

“From the moment we settled in this place, we were clear that we were in a risk zone. That’s precisely why we always carried out mitigation work ourselves, as neighbors—we cleaned the ravines so they wouldn’t fill with trash. We want to keep the ravine channel clear so it doesn’t affect us.” (Settlement leader, 2024)

However, these capacities have structural limits: families’ precarious economic situation, and conditions of access to water in terms of cost and quality, along with dampness inside homes, expose families to various illnesses. In addition, living in extractive mining cities restricts the everyday possibility of minimizing the condition of vulnerability in which they live.

### 5.2 The negotiated and relational character of community resilience

The evidence from the Andacollo sector allows us to question romanticized visions of community resilience that implicitly transfer responsibility for adaptation onto communities themselves. As Figure 4 shows, bonding social capital (housing committees, WhatsApp alert networks, collective ravine cleaning) enables a partial, everyday reduction in risk exposure, but does not by itself constitute a pathway to transformative resilience: internal cohesion cannot substitute for unequal access to infrastructure, urbanization, potable water, and mitigation works. This finding is consistent with Manyena et al.'s (37) distinction between a capacity to “bounce back” and a capacity to “bounce forward,” and with Folke et al. (33) argument that transformative resilience requires the creation of new structural conditions, not merely adaptation to existing ones. Community resilience in Andacollo thus reveals itself as a negotiated, relational process, unequally distributed across sectors of the same territory, rather than as a homogeneous or intrinsic attribute of “the community.”

Read through the distinction between coping, adaptive, and transformative resilience (33, 45), the data allow us to more precisely identify what actually transforms the Andacollo community and makes it resilient, and which elements would need to be strengthened. What everyday transforms risk exposure into a capacity for collective action is, first, the conversion of repeated experience of hazard into shared technical knowledge—the periodic cleaning of ravines and neighborly monitoring described by the leaders are not merely coping practices but a form of accumulated territorial learning that Berkes and Ross (35) directly associate with local agency. Second, the densification of informal early-warning networks—the WhatsApp groups mentioned in the workshops—transforms scattered information into a distributed surveillance system that reduces reaction time to flash-flood events, even though it does not alter structural exposure. Third, the existence of formally constituted housing committees transforms informal neighborhood organization into a recognizable interlocutor for institutions, a necessary—though not sufficient—condition for accessing linking capital. However, none of these three mechanisms alone reaches the threshold of transformative resilience, as reinforced in Figure 4.

### 5.3 Risk communication, sanitation, and the social determinants of health

The health findings from the go-along walks—reflected mainly in greywater flows, wastewater discharge into flash-flood evacuation channels, reliance on septic pits without regular emptying, and groundwater contamination—should not be read solely as conditions of deficient infrastructure, but as territorial expressions of the social determinants of health: the unequal distribution of sanitation, potable water, and safe electrification between registered and unregistered settlements configures a pattern of health inequity that overlaps with, and is frequently conflated with, exposure to mass movement and structural fire. Taken together, neighborhood WhatsApp alert networks—activated in response to flash floods and fires as well as to health risks—constitute a community risk-communication infrastructure that operates, de facto, at the intersection of disaster risk reduction and territorial public health, even though it remains invisible to institutional epidemiological surveillance and early-warning systems. Strengthening this informal infrastructure, and explicitly linking it to sanitation protocols, would help overcome a form of risk communication that currently treats health and geomorphological hazards as separate, when fieldwork suggests that communities experience both dimensions as a single condition of territorial vulnerability. To this must be added the climatic conditions specific to the Atacama Desert—intense nighttime cold and dampness in homes built of precarious materials—the lack of access of older adults with reduced mobility to the public health network given the elevated location of the settlements, and an unaddressed mental health burden that worsens among the migrant population in irregular status, who avoid contact with formal services for fear of institutional exposure. This set of findings positions the Andacollo sector as a paradigmatic case of interlinked social determinants of health—climate, mobility, mental health, and migratory status—and not merely as a case of geomorphological risk exposure.

## 6 Conclusion

Informal settlements in Copiapó demonstrate that disaster risk does not constitute an exceptional condition tied exclusively to socio-natural hazards, but rather the cumulative result of processes of unequal urbanization, housing exclusion, and institutional fragmentation. The expansion of settlements into zones exposed to mass movement and excluded from urban planning reflects how differential access to land and housing continues to displace lower-income populations toward environmentally vulnerable territories. Risk, therefore, does not operate as an element external to informality but as one of its constitutive dimensions. The results show that social capital constitutes a fundamental resource for sustaining forms of community resilience in contexts of territorial precariousness. Everyday solidarity networks, neighborhood organization, and community coordination have enabled communities to confront health crises, fires, and flash-flood hazards through adaptive mechanisms built from within the territory itself. However, not every form of resilience implies structural transformation (45).

Three interconnected conclusions emerge from this study. First, bonding social capital—expressed in neighborhood committees, care networks, WhatsApp alert systems, and collective strategies for addressing health and flash-flood hazards—enables a partial reduction of immediate risk exposure, but has structural limits: internal social cohesion cannot replace unequal access to infrastructure, urbanization, potable water, sanitation, or mitigation works. Second, linking social capital constitutes a decisive element for understanding inequalities in the production of community resilience. Communities with greater institutional articulation gain access to urbanization, mitigation, and regularization processes that sustainably reduce their vulnerability; settlements excluded from cadastral registries and formalization processes remain trapped in structural precariousness. Third, transformative resilience depends critically on the capacity to modify the institutional and territorial structures that produce vulnerability. Bureaucratic fragmentation, regulatory limits, and inequalities in institutional access continue to constrain the possibilities for structural transformation of risk in the settlements studied.

These findings have direct practical implications. First, public agencies should formally recognize and resource the bonding-capital infrastructure that already exists—such as neighborhood WhatsApp alert networks and the coordination of housing committees—rather than treating it as an autonomous substitute for state investment, explicitly integrating it into municipal and regional early-warning and risk-communication protocols. Second, access to mitigation works, regularization, and basic services should be decoupled from cadastral and concession status, and instead prioritized according to actual multi-hazard exposure, so that linking capital no longer remains a scarce resource distributed according to bureaucratic seniority. Third, preparedness for structural fires and the treatment of physical and mental health issues require the same inter-institutional coordination architecture currently reserved for flash-flood risk, given their comparable lethality and their current institutional neglect. A gender-responsive resilience policy should channel resources toward reducing women’s unremunerated burden of care and coordination—for example, through funded community leadership roles—rather than treating their organizational work as a cost-free input to resilience.

In sum, advancing toward effective disaster risk reduction strategies in informal settlements requires moving beyond emergency-centered approaches toward preventive, intersectoral, and inequality-sensitive territorial policies. The articulation among SENAPRED, SERNAGEOMIN, MINVU, SERVIU, the Precarious Settlements unit, the DOH, and the Municipality of Copiapó—together with Andacollo’s community leaders—constitutes a first requirement for transitioning from reactive resilience toward the construction of communities that are aware of and prepared for any potential hazard event. In extractive cities such as Copiapó, where informal urbanization continues to expand over territories of high environmental exposure, strengthening more equitable forms of institutional articulation is an indispensable condition for advancing toward genuinely transformative processes of resilience.

## Data Availability

The raw data supporting the conclusions of this article will be made available by the authors, without undue reservation.
